# Intravascular large B-cell lymphoma arising in the pituitary gland: A case report

**DOI:** 10.1097/MD.0000000000040995

**Published:** 2024-12-13

**Authors:** Kyu Yun Jang, Ae Ri Ahn

**Affiliations:** a Departments of Pathology, Jeonbuk National University Medical School, Research Institute of Clinical Medicine of Jeonbuk National University, Biomedical Research Institute of Jeonbuk National University Hospital, and Research Institute for Endocrine Sciences, Jeonju, Jeonbuk, Republic of Korea.

**Keywords:** immunohistochemistry, lymphoma, pituitary gland

## Abstract

**Rationale:**

Primary pituitary lymphoma is defined as a lymphoma that develops only in the pituitary gland without involvement of other areas.

**Patient concerns:**

We present the case of a 61-year-old female patient who underwent an endonasal transsphenoidal approach for the preoperative diagnosis of a pituitary macroadenoma based on radiological findings.

**Diagnoses:**

Microscopically, the capillaries were distended by tumor cells. The high-magnification view showed large cells with vesicular nuclei and single or multiple prominent nucleoli, with mitotic figures often observed within the intravascular space.

**Interventions:**

Immunohistochemical staining showed strong positivity for CD45 and pan-B cell markers such as CD19 and CD20. The postoperative diagnosis was intravascular large B-cell lymphoma of the pituitary gland.

**Outcomes:**

Next-generation sequencing revealed alterations in 12 genes: *ARID5B*, *BCL2*, *CD79B*, *ETV6*, *HLA-B*, *LRRC7*, *MYD88*, *PIM1*, *POT1*, *PTPN11*, *RASA1*, and *SRSF2*.

**Lessons:**

To our knowledge, this is the first case report of pituitary intravascular large B-cell lymphoma.

## 1. Introduction

Primary pituitary lymphoma (PPL) is defined as a lymphoma that develops only in the pituitary gland without involvement of other areas at the time of diagnosis. PPL is an extremely rare entity, with 57 reported cases to date, and constitutes 0.2% of pituitary lesions. Diffuse large B-cell lymphoma is the most common type of PPL.^[1,2]^ To date, there have been no reports of intravascular large B-cell lymphoma of the pituitary gland. Although radiological techniques such as computed tomography (CT) and magnetic resonance imaging (MRI) are useful for the differential diagnosis of pituitary lesions, preoperative diagnosis without biopsy is difficult because the clinicopathological features of PPL have not yet been fully clarified. Here, we report the case of a female patient with intravascular large B-cell lymphoma arising in the pituitary gland whose radiological findings mimicked those of a pituitary macroadenoma. In addition, we briefly reviewed the literature on the clinical and histopathological features of PPL.

## 2. Case presentation

**Figure 1.**
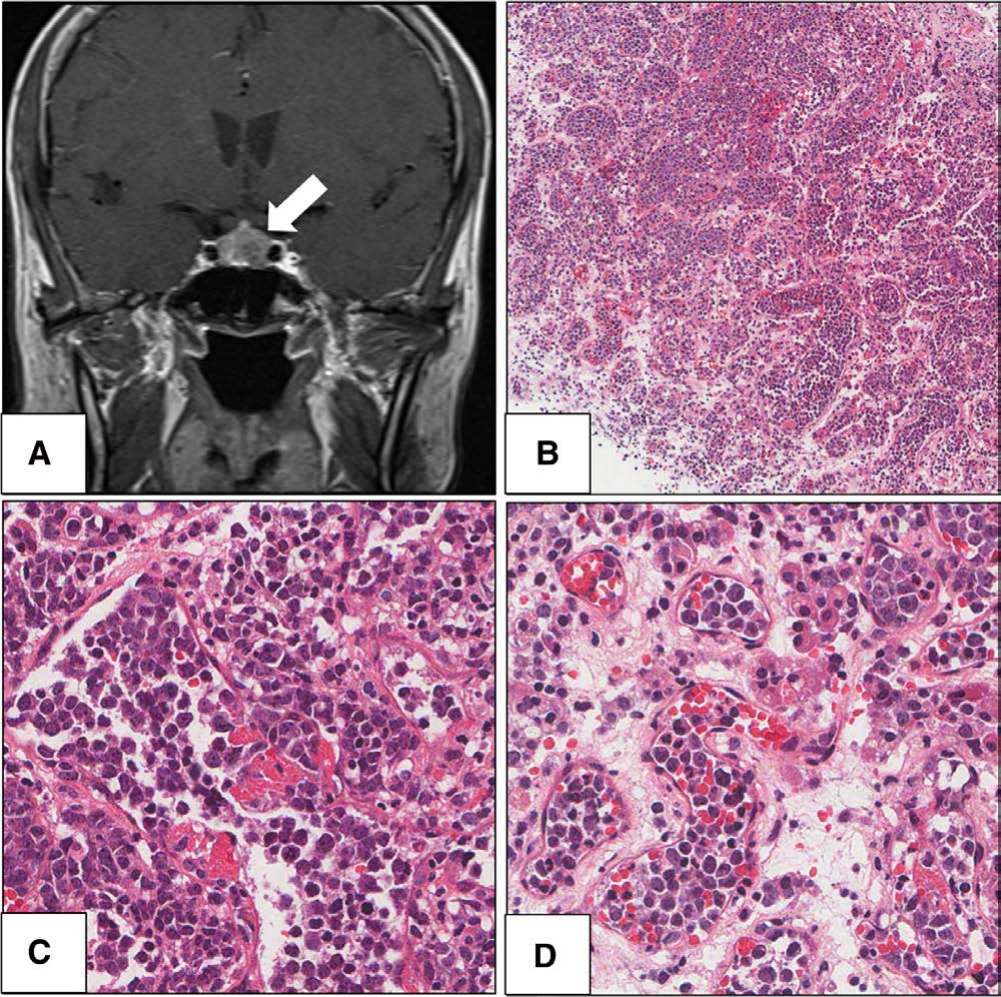
Radiological findings and histopathological of intravascular large B-cell lymphoma arising in a pituitary gland. (A) Magnetic resonance imaging demonstrated a 1.2-cm-sized mass in pituitary gland (hematoxylin and eosin (H & E), ×100). (B) Microscopic findings revealed capillaries distended by tumor cells. (C) In higher-magnification, large sized cells with vesicular nuclei and single or multiple prominent nucleoli were observed (H & E, ×400). (D) Tumor cell clusters are observed within the lumina of small vessels (H & E, ×400).

A 61-year-old female patient presented to the neurosurgery department with a 3-week history of severe headache and weight loss of 5 kg. The patient had no relevant medical history. Physical examination revealed no neurological symptoms such as blurring of vision or visual field defects. Laboratory examinations were as follows: follicle-stimulating hormone, 1.3 mIU/mL (normal range: 1.4–18.1 mIU/mL); luteinizing hormone, <0.1 mIU/mL (normal range: 1.5–9.3 mIU/mL); prolactin, 26.5 ng/mL (normal range: 2.1–17.7 ng/mL); thyroid stimulating hormone, 0.06 mIU/mL (normal range: 0.17–4.05 mIU/mL); free T4, 12.0 pmol/L (normal range: 11.5–23 pmol/L); total triiodothyronine, 0.95 ng/mL (normal range: 0.83–1.87 ng/mL); adrenocorticotropic hormone, 16.8 pg/mL (normal range: 7.2–63.3 pg/mL); antidiuretic hormone, 2.0 pmol/L (normal range: 0–13.0 pmol/L); and serum cortisol (at 8:00 am), 24.1 μg/dL (normal range: 4.3–22.4 μg/dL). CT of the paranasal sinuses revealed nonspecific findings with no abnormal mucosal thickening or mass lesions in the paranasal sinuses, no evidence of a mass in the nasal cavity, and no evidence of bone erosion or destruction. For further evaluation, the patient underwent sellar MRI. Contrast-enhanced MRI revealed a heterogeneously enhancing mass. The mass size was 1.2 × 1.2 × 1.0 cm (Fig. 1A). 2-[Fluorine-18]-fluoro-2-deoxy-D-glucose-positron emission tomography/CT showed increased FDG uptake in the pituitary gland without systemic uptake. Based on these clinical findings, the final diagnosis was pituitary macroadenoma. Hence, the patient underwent an endonasal trans-sphenoidal approach for diagnosis and treatment.

**Figure 2.**
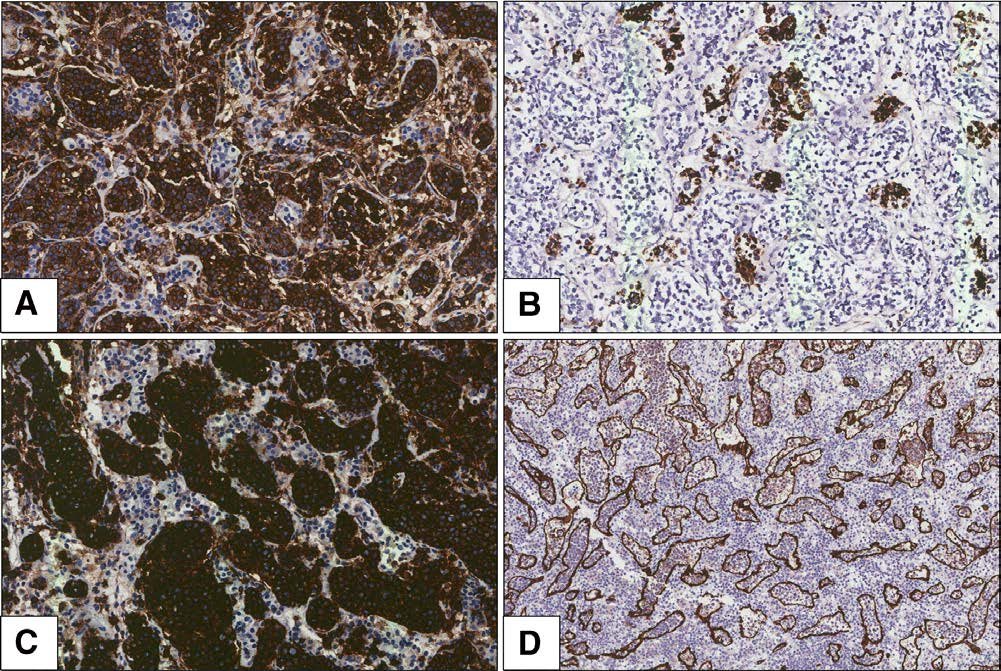
Immunohistochemical findings of intravascular large B-cell lymphoma arising in a pituitary gland. (A) Tumor cells exhibited immunoreactivity for CD45 (original magnification, ×200). (B) There was also a focal positivity of expression of pan-cytokeratin on neoplastic cells (original magnification, ×200). (C) Tumor cells exhibited immunoreactivity for CD20 (original magnification, ×200). (D) CD34 immunohistochemistry highlighting large B cells restricted to intravascular spaces (original magnification, ×100).

Gross examination of the specimen revealed grayish-white hard tissue measuring 1.2 × 0.7 cm. Microscopic examination of the resected specimen revealed capillaries distended by tumor cells (Fig. 1B). The high-magnification view showed large cells with vesicular nuclei and single or multiple prominent nucleoli, and mitotic figures were often observed within the lumina of the small vessels (Fig. 1C, D). Immunohistochemistry was performed to identify the tumor cell lineage. Immunohistochemical staining revealed strong positivity for CD45 and negativity for CD138 and myeloperoxidase (Fig. 2A). Focal positivity was observed for pan-cytokeratin (CK) expression in neoplastic cells with expression of other keratins (CK5/6, CK7; Fig. 2B). Tumor cells exhibited immunoreactivity for CD19 and CD20 and were negative for CD3 (Fig. 2C). CD34 immunohistochemistry revealed large B cells restricted to the intravascular spaces (Fig. 2D). Based on these findings, the possibility of CK-positive malignant B-cell lymphoma was suggested. In addition, immunohistochemical staining was positive for CD5, BCL2, and BCL6 with focal positivity observed for MUM-1. Tests for anaplastic lymphoma kinase, c-MYC, cyclinD1, CD10, CD30, and terminal deoxynucleotidyl transferase were negative. In situ hybridization results were negative for Epstein-Barr virus-encoded RNA. Ki-67 was strongly expressed in tumor cells (70%). Pathological diagnosis confirmed that the mass was an intravascular large B-cell lymphoma arising in the pituitary gland.

We performed targeted next-generation sequencing panel testing with the Oncomine Comprehensive Assay Plus (Thermo Fisher Scientific, Waltham) using resected specimens. The result showed 12 single nucleotide variations: *ARID5B* (c.2554C > T, p.Gln852Ter), *BCL2* (c.256C > G, p.Leu86Val), *CD79B* (c.590A > G, p.Tyr197Cys), *ETV6* (c.26G > A, p.Ser9Asn), *HLA-B* (c.809C > T, p.Ala270Val), *LRRC7* (c.4028C > T, p.Ser1343Leu), *MYD88* (c.755T > C, p.Leu252Pro), *PIM1* (c.577C > T, p.Leu193Phe), *POT1* (c.190G > T, p.Gly64Ter), *PTPN11* (c.1445A > G, p.Lys482Arg), *RASA1* (c.1211C > T, p.Thr404Met), and *SRSF2* (c.341G > A, p.Gly114Asp) variants. This case report was approved by the Institutional Review Board of the Jeonbuk National University Hospital (approval no. IRB 2024-08-018). Informed consent was obtained from the patient for publication.

## 3. Discussion and conclusion

PPL is a rare entity that shows involvement only in the pituitary gland, without systemic involvement. Most PPL cases are non-Hodgkin’s lymphomas. Diffuse large B-cell lymphoma is the most common PPL, and other subtypes include mucosa-associated lymphoid tissue lymphoma and T-cell lymphoma. According to previous studies, the average age at PPL onset is approximately 60 years, with a slight female predominance. The common clinical presentations of PPL are headache, hypopituitarism, diplopia, and hemianopia.^[3,4]^ PPLs show no specific radiological findings. Radiologically, PPLs are difficult to differentiate from other pituitary neoplasms. To date, no case of intravascular large B-cell lymphoma with the pituitary gland as the primary site has been reported.

Intravascular large B-cell lymphoma is an aggressive subtype of extranodal B-cell lymphoma, characterized by the proliferation of large neoplastic B cells virtually exclusively within the lumen of blood vessels.^[5]^ The prognosis of intravascular large B-cell lymphoma is poor because of delayed diagnosis and an aggressive course. Chemotherapy with rituximab is a well-known treatment for intravascular large B-cell lymphoma. In cases with central nervous system involvement, methotrexate-based chemotherapy is effective in improving patient outcomes.^[6]^ Differential diagnosis of pituitary intravascular large B-cell lymphoma is challenging. The initial impression in the present case masqueraded as a pituitary macroadenoma.

Histopathological findings revealed large lymphoid cells with vesicular nuclei and single or multiple prominent nucleoli within the lumen of small vessels, especially capillaries. Mitotic figures were often observed. In addition, free-floating, cohesive, and marginal growth patterns were observed. The identification of neoplastic lymphoid cells restricted to intravascular spaces is an essential diagnostic criterion for intravascular large B-cell lymphoma. Large B cells restricted to intravascular spaces were highlighted by immunohistochemistry using endothelial markers such as CD31 and CD34. Intravascular large B-cell lymphoma shows the immunophenotype of pan-B-cell markers such as CD19 and CD20.^[6]^ The present case is consistent with previous reports. However, in the present case, CK expression was difficult to confirm and delayed the diagnosis. To the best of our knowledge, this is the second case of CK expression in an intravascular large B-cell lymphoma.^[7]^

In conclusion, intravascular large B-cell lymphoma of the pituitary gland is an extremely rare and incidental finding during pathological examination. Pathologists should be aware of this condition to avoid its misdiagnosis as pituitary adenoma. Herein, we presented a case of intravascular large B-cell lymphoma of the pituitary gland that mimicked as pituitary macroadenoma. This is the first case report of intravascular large B-cell lymphoma arising in the pituitary gland. This case report will help clarify the clinicopathological characteristics of intravascular large B-cell lymphoma of the pituitary gland and establish a precise differential diagnosis. Further evaluation, including molecular studies, can help in the development of targeted therapies.

## Acknowledgments

This paper was supported by Fund of Biomedical Research Institute, Jeonbuk National University Hospital and Korea Health Technology R&D Project through the Korea Health Industry Development Institute (KHIDI), funded by the Ministry of Health & Welfare, Republic of Korea: HR22C1832.

## Author contributions

**Conceptualization:** Kyu Yun Jang, Ae Ri Ahn.

**Data curation:** Kyu Yun Jang, Ae Ri Ahn.

**Investigation:** Ae Ri Ahn.

**Methodology:** Kyu Yun Jang, Ae Ri Ahn.

**Project administration:** Kyu Yun Jang, Ae Ri Ahn.

**Writing – original draft:** Kyu Yun Jang, Ae Ri Ahn.

**Writing – review & editing:** Kyu Yun Jang, Ae Ri Ahn.

## References

[1] Lam AK. Pathology of endocrine tumors update: World Health Organization new classification 2017—other thyroid tumors. AJSP Rev Rep. 2017;22:209–16.

[2] Caputo M Nunzia P Alessandro B. Primary pituitary lymphoma as rare cause of a pituitary mass and hypopituitarism in adulthood. Endocr Pract. 2020;26:1337–50. 33471665 10.4158/EP-2020-0286

[3] Cai S Xiao J Chen P Luo H Cheng Z. Primary pituitary stalk mucosa-associated lymphoid tissue lymphoma: a case report and literature review. Front Neurol. 2023;14:1193391. 37554391 10.3389/fneur.2023.1193391 PMC10406508

[4] Sreedhara P Czerwinski V Alexander E Choudhary C. T-cell lymphoblastic lymphoma presenting as a pituitary mass. JCEM Case Rep. 2023;1:97. 10.1210/jcemcr/luad097 PMC10580472 37908217

[5] Ong YC Kao HW Chuang WY. Intravascular large B-cell lymphoma: a case series and review of literatures. Biomed J. 2021;44:479–88. 32344119 10.1016/j.bj.2020.04.005 PMC8514799

[6] Roditi E Panicker S Fung AT. Intravascular large B-cell lymphoma of the eye: literature review and new findings. Asia Pac J Ophthalmol (Phila). 2024;13:100053. 38556129 10.1016/j.apjo.2024.100053

[7] Coulibaly B Mesturoux L Petit B Magy L Labrousse F. Intravascular large B-cell lymphoma presenting as cauda equina syndrome and showing aberrant cytokeratin expression: a diagnostic challenge. Pathology (Phila). 2014;46:241–4. 10.1097/PAT.0000000000000089 24614706

